# Lifestyle interventions for patients with non-alcoholic steato-hepatitis–Design, rationale and protocol of the study “target group-specific optimisation of lifestyle interventions for behavior change in non-alcoholic steato-hepatitis (OPTI-NASH)”

**DOI:** 10.1371/journal.pone.0288905

**Published:** 2023-07-27

**Authors:** Kathrin Krüger, Carina Oedingen, Achim Kautz, Diane Langenbacher, Siegbert Rossol, Jona T. Stahmeyer, Christian Krauth

**Affiliations:** 1 Institute for Epidemiology, Social Medicine, and Health Systems Research, Hannover Medical School (MHH), Hannover, Germany; 2 Center for Health Economics Research Hannover (CHERH), Hannover, Germany; 3 Erasmus School of Health Policy & Management, Erasmus University Rotterdam, Rotterdam, The Netherlands; 4 Kautz^5^, Cologne, Germany; 5 Department of Internal Medicine, Krankenhaus Nordwest, Frankfurt on Main, Germany; 6 Health Services Research Unit, AOK Niedersachsen, Hannover, Germany; UNITED KINGDOM

## Abstract

**Background:**

Non-alcoholic steato-hepatitis (NASH) is the inflammatory, progressive form of non-alcoholic fatty liver disease (NAFLD). A delayed diagnose interval is typical for the majority of the patients because of the asymptomatic natural course. However, serious sequelae may develop such as cirrhosis or hepatocellular carcinoma. NASH is also associated with an increased risk of metabolic diseases. Obesity developed due to a lack of exercise or a disadvantageous diet often leads to NAFLD or NASH, thereby interventions including enhanced physical activity and calorie reduction form the actual gold standard of treatment. To date, patients rarely use these. The project aims to model lifestyle interventions based on the preferences of the NASH patients.

**Methods:**

Based on a systematic review and focus group discussions, two discrete choice experiments (DCE) will be designed, one on aspects influencing successful uptake of lifestyle interventions and one to analyses parameters contributing to long-term participation. An online survey will be used to elicit patient’s preferences on program design and on motivational aspects in a cross-sectional design. The recruitment will take place in nine certified specialist practices and hospital outpatient clinics aiming to reach a sample size of n = 500 which is also required for the DCE design.

**Discussion:**

The results will provide an overview of the NASH patient’s preferences regarding the successful uptake and long-term implementation of lifestyle interventions. Recommendations for optimized lifestyle change programs will be derived and an intervention manual will be developed to facilitate target group-specific inclusion in programs in practice.

## Introduction

Non-alcoholic steato-hepatitis (NASH) is the inflammatory and progressive form of non-alcoholic fatty liver disease (NAFLD). Due to non-specific symptoms, a majority of patients remain undetected until serious sequelae occur, such as liver fibrosis, liver cirrhosis, or hepatocellular carcinoma (HCC). NASH is also associated with an increased risk of type 2 diabetes mellitus (DM2), cardiovascular disease, and obesity [1]. However, according to the national guideline, there is no drug therapy currently available to reduce NASH [2]. Current data on the burden of disease of NAFLD in Western industrialized nations show that there were approximately 18.4 million patients in Germany in 2016, with an expected increase to 20.9 million by 2030 [3]. Many of them are not yet diagnosed and therefore are not informed about the potentially chronic and progressive nature of fatty liver disease. In particular, the predicted increase in potentially progressive NASH patients from 3.3 million in 2016 to up to 4.7 million in 2030 points to the challenges faced by these patients, who lack a current treatment alternative [3]. Due to their growing prevalence, NAFLD and NASH are increasingly establishing themselves as a public health challenge. NASH is often caused by obesity resulting from a lack of physical activity or a disadvantageous diet. Therefore, global, European, and national studies have shown that guided lifestyle interventions can have a positive effect on the course of NASH and can significantly reduce the burden on the health care system [4]. For example, interventions aimed at reducing body weight have led to an improvement in histological liver changes [4–6]. Lifestyle interventions are particularly relevant for patients with pre-existing fibrosis, as they would benefit directly from a possible regression of their fibrosis. Unfortunately, these interventions are rarely taken up by these patients [7]. Therefore, the particular challenge is that patients must increasingly assume personal responsibility in order to avoid the progression of NAFLD/NASH based on a healthier lifestyle and to prevent consequences such as cirrhosis, liver transplantation, or HCC.

Moreover, lifestyle interventions can only be successful when they are implemented in a sustainable and long-term way. There is a consensus that existing offers for lifestyle interventions (e.g., weight reduction, exercise activation, healthy nutrition) are heterogeneous, but that they are not sufficiently accepted by patients with a high medical need or they are just accepted by patients with high health literacy and behavioral affinity. Previous analyses have shown that a lack of knowledge and understanding of the disease often contributes to the fact that these patients do not see the need to change their lifestyle behavior [8]. Therefore, for a sufficient acceptance of lifestyle changes, it is necessary that these interventions have to be oriented towards the preferences of the patients. This also aims that patients are motivated and activated to change their lifestyle behavior and to overcome the lack of knowledge about their own disease. The current offers do not often fulfil the criteria of the target group [7].

## Materials and methods

### Study aim and setting

The project aims to improve care by optimizing the provision of target group-specific lifestyle interventions by designing programs according to patients’ preferences in order to ensure successful and long-term participation and thus initiate sustainable lifestyle changes. Based on the findings, the project aims to generate recommendations for optimized lifestyle change programs as well as program contents that contribute to the successful implementation of lifestyle change. The improved programs should take into account the specifics of the subgroups and the clinical context. In addition, the preferences of NASH patients will be compared with the programs identified in the course of the research in order to find out whether suitable programs already exist for certain target groups. Furthermore, an intervention manual for specialist practices or outpatient clinics will be developed for the target-group-specific steering of NASH patients as well as for the successful implementation of suitable lifestyle interventions. The project is divided into three work packages (WP), to which the following research questions are assigned:

#### WP 1: Systematic overview

- Which current programs are existing?
- Are there already programs that meet the preferences of specific target groups?
- How are best practice examples (including international examples) designed and which parameters lead to successful implementation?

#### WP 2: Online survey including Discrete Choice Experiments (DCE)

- What knowledge do NASH patients have about their disease?
- How motivated are NASH patients to change their lifestyle (in terms of physical activity and dietary behavior)? What determinants influence their motivation?
- Have the surveyed NASH patients already used lifestyle interventions and what barriers did they encounter?
- What preferences for lifestyle interventions do NASH patients have? What are the preferences of those who have already used lifestyle interventions?

#### WP 3: Deliverables

- How can access and successful implementation be optimized or tailored for different subgroups?
- How should target group-specific lifestyle interventions be designed and what components should they contain?

### Study design

**Fig 1:**
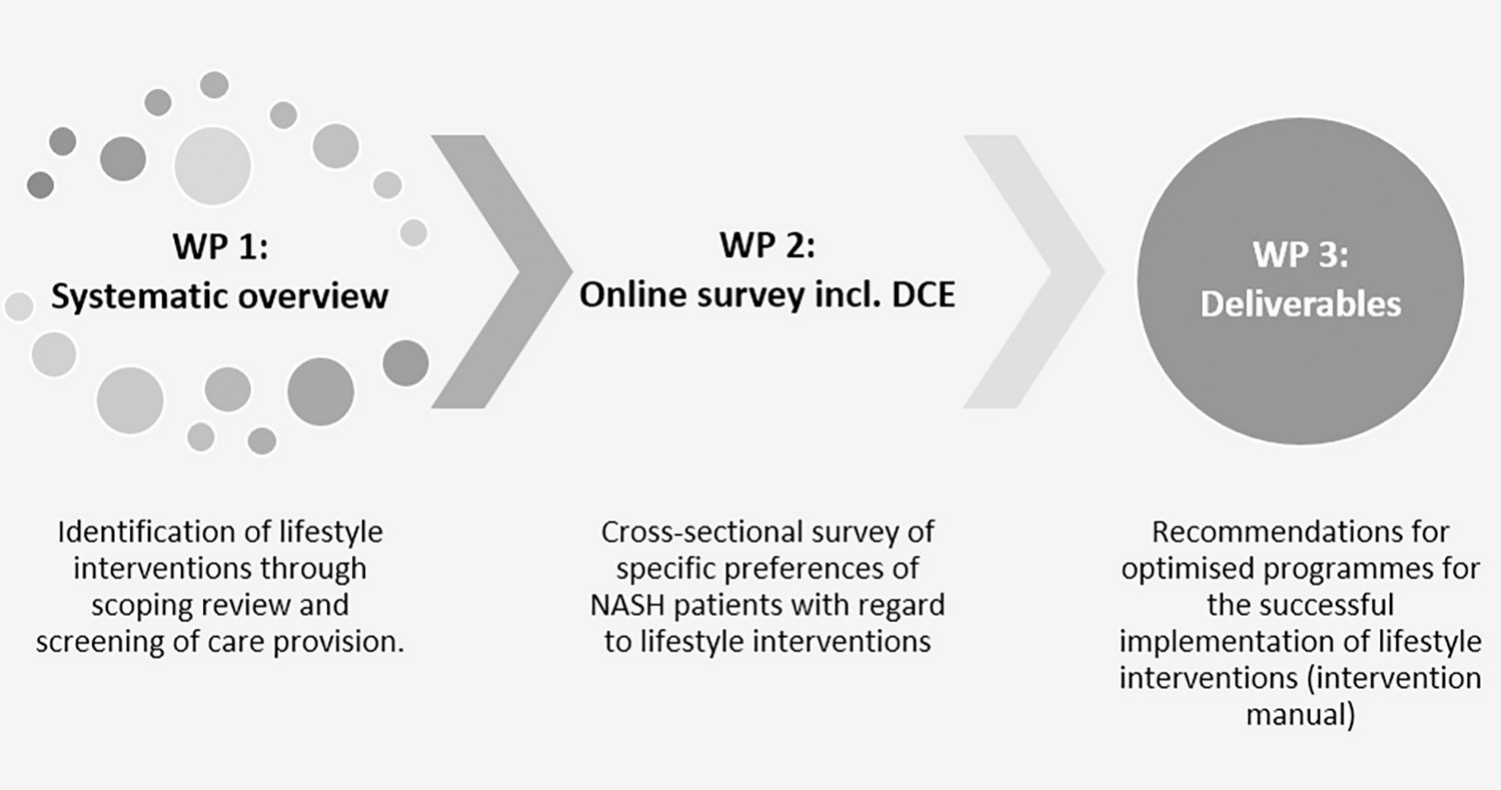
Study design.

The project is divided into three WP according to the research questions (Fig 1): (1) First, lifestyle interventions are researched based on a scoping review and a screening of current available intervention programs. Characteristics of effective programs that have high participation rates and low dropout rates will be extracted. (2) Based on this, two Discrete Choice Experiments (DCE) will be developed, one on program design and one on motivation. In an online survey, knowledge, motivation, experiences, and preferences of a representative patient sample will be quantitatively assessed. The DCEs serve to examine which lifestyle interventions are preferred by the patients and which aspects are relevant to decision-making. (3) In the last step, recommendations for components of an optimized lifestyle change program, which takes into account the preferences of the subgroups and the clinical context from a hepatological perspective, will be derived from the generated data. Components will be defined that facilitate the target group-specific integration of patients into programs as well as that ensure the continuous participation of the subgroups.

#### WP 1: Systematic overview

As part of WP 1, existing programs as well as best practice examples (including international examples) are to be identified. For this purpose, a scoping review will first be conducted in relevant databases using a predefined search strategy. The research should identify relevant national and international publications. The project group will independently review the research results.

In a second step, current programs will be examined in order to identify existing programs and best practice examples that are successful in practice, but for which there may not yet be any results reported or even published. A standardized questionnaire will be developed within the project to record the programs and best practice examples. This questionnaire will contain questions about which services are known or implemented in relation to NASH patients and what kind of interventions (e.g., dietary behavior, physical activity) are involved. In order to be able to represent a broad spectrum various institutions will be interviewed: experts in certified specialist practices and hospital outpatient clinics; specialist societies and professional associations; patient support groups; the twenty largest health insurance funds in Germany as well as international networks.

#### WP 2: Online survey including DCE

**Fig 2:**
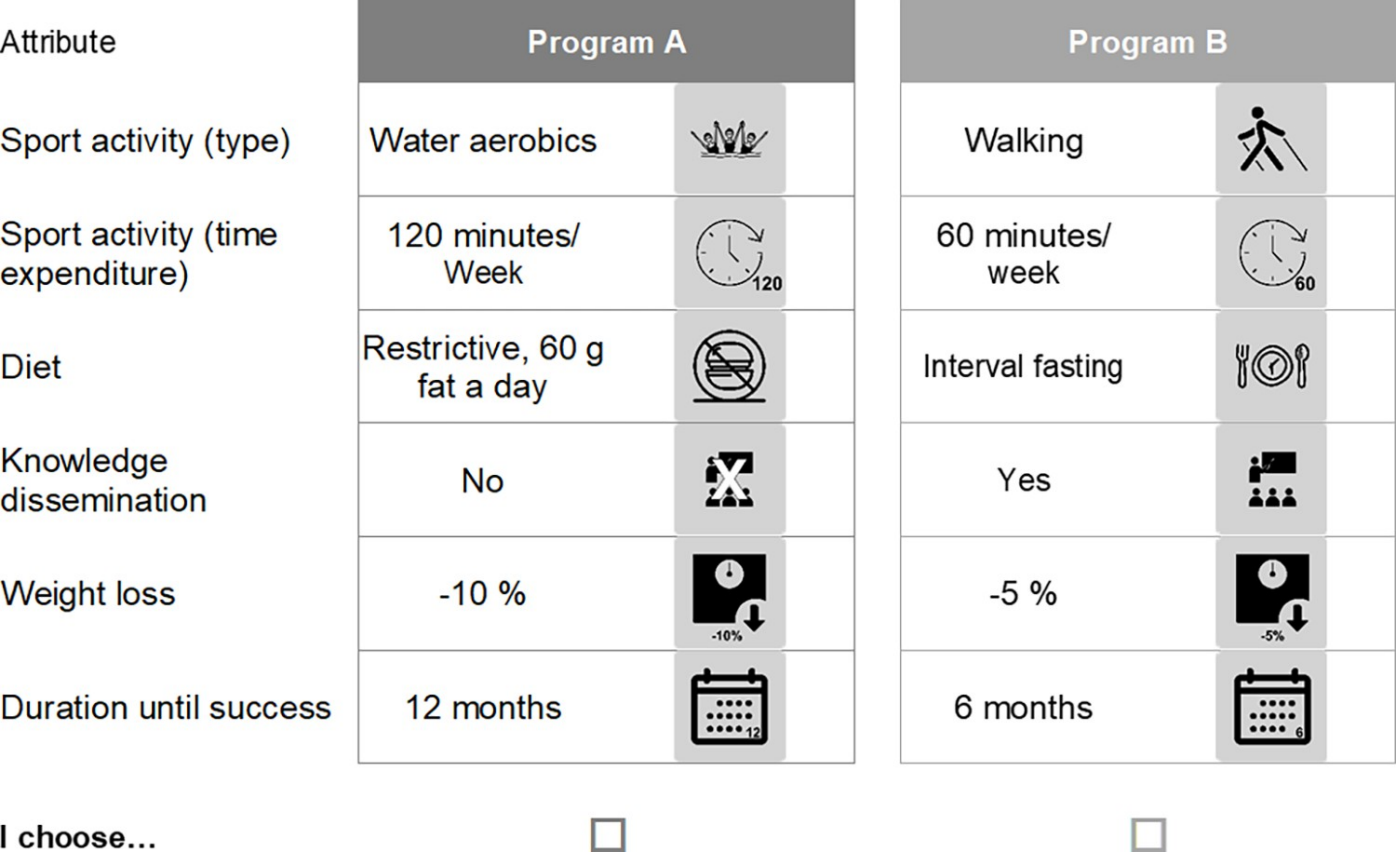
Example for a choice set regarding program design.

**Fig 3:**
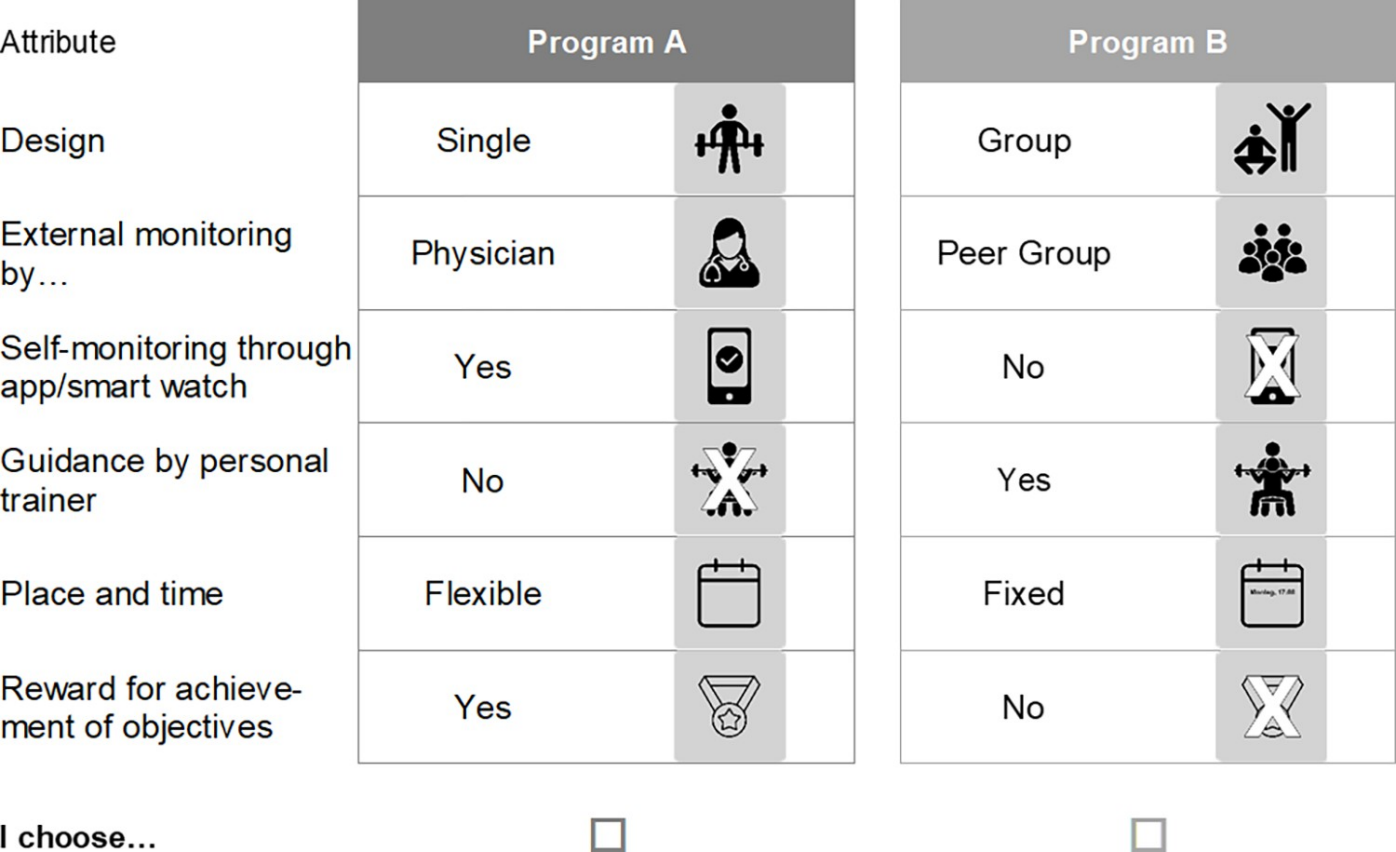
Example for a choice set regarding motivational aspects.

In WP 2, preferences will be collected using a self-complete online questionnaire. The questionnaire will measure knowledge, motivation, experiences, preferences, etc. of the defined target population. The main element of the questionnaire will be the two DCEs. One DCE is to identify aspects that influence successful uptake of a lifestyle intervention, i.e., how a program must be designed for NASH patients to decide to participate. Aspects such as the type and duration of physical activity, the type of diet/nutritional changes, and the provision of knowledge could be influential here (Fig 2). The second DCE will address the motivation of NASH patients concerning lifestyle interventions, i.e., what aspects lead to their long-term participation in a program. Program design, monitoring, guidance, or rewards for achieving objectives could have an impact on motivation to participate long-term (Fig 3).

The experimental method of discrete choice modelling has already been applied in health economics since the 1990s and is increasingly used to evaluate preference-sensitive decisions in health care [9]. For this purpose, hypothetical choice scenarios (so-called choice sets) with different options are designed, which are composed of the characteristics (attributes) of the options and their variations (levels). From each of two or three alternatives, the option that is more preferred, i.e. has the higher utility for the respondents, is to be selected (Figs 2 and 3). This is repeated up to eight times with differently choice sets. The analysis of the choices provides information about the preferred attributes and their levels. Therefore, choice decisions can also be simulated realistically and individual attributes analyzed, so that latent preference structures of the respondents can be revealed and the trade-offs between different attributes can be determined [9]. In accordance with the recommendations of the Conjoint Analysis Checklist (International Society for Pharmacoeconomics and Outcomes Research (ISPOR)) [10], the selection.of decision-relevant attributes and their levels will be carried out by a) a systematic review and b) focus group discussions with patients.

The design of the final choice sets (design) is based on the final number of attributes and levels and can therefore not be determined in advance. However, as the number of possible combinations and thus the number of choice sets increases with the number of attributes and levels, a full factorial design would demand high cognitive abilities from the respondents and will also take a lot of time. For example, with 3 attributes with 3 levels, 1 attribute with 4 levels and 2 attributes with 2 levels, a total of 33*41*22 = 432 possible combinations and thus 432 choices would be available. Therefore, a reduced (fractional factorial) design [11–13] will be chosen and, if necessary, will be divided into blocks. Thus, the respondents will be presented with only one block with 8 choice sets, i.e., a subset of the entire design. In addition, a dominant choice set (so-called no-brainer) will be added to each block to check for internal validity and plausibility. A d-efficient design will be calculated using the SAS OnDemand for Academics software (SAS Institute, Cary, NC, USA).

First, a systematic review will be conducted. For this purpose, relevant databases will be searched using a predefined search strategy that includes different search terms on NASH, preferences and lifestyle interventions. The search will identify relevant national and international publications on the preferences of NASH patients regarding lifestyle interventions. Two researchers will independently screen titles, abstracts and full texts according to predefined inclusion and exclusion criteria.

Second, four focus group discussions with 6–8 participants each will be conducted. As far as possible, half of the participants should have experience in lifestyle interventions and the other half should have no experience. In addition, the groups should be homogeneous in terms of disease duration (incidence/prevalence). No composition will be specified with respect to age and gender. Participants will receive an expense allowance of 50 euros. Recruitment will be done through certified specialist practices and hospital outpatient clinics. Using a guideline to be developed, different factors will be discussed regarding their importance for the decision for or against different lifestyle interventions. Group discussions will be scheduled for a period of 90–120 minutes.

Third, together with the results of the systematic research, the attributes for the two DCEs will be selected and the corresponding levels for the different choices will be elaborated. The selected attributes and their levels will be combined into different choice sets. The goal is a maximum of 6–8 choice sets per respondent per DCE, which will be inserted into the questionnaire.

In addition to the DCE, the questionnaire will include, inter alia, items related to the disease, lifestyle interventions, health-related parameters, socio-demographic and socio-economic parameters of the target population. With regard to lifestyle interventions, previous experiences, hurdles and barriers encountered, and motivating and demotivating factors will be recorded. With regard to the disease, it will be ascertained when it was diagnosed and what therapeutic measures were taken or what recommendations were made. In addition, self-assessed knowledge, information needs, and information behavior, as well as disease knowledge and disease perception, will be recorded. To generate different subgroups, for example, health-related parameters (general health, height, weight (to derive BMI), chronic diseases, quality of life), motivational aspects and aspects of health behavior or lifestyle (diet, sport/exercise, alcohol, smoking, drugs), as well as socio-demographic (age, gender, etc.) and socio-economic parameters (education, income, etc.) will be recorded. Various standardized survey instruments will be used to collect certain parameters, including:

- Quality of life: EuroQol 5D (EQ-5D) [14].
- General health status: WHO Disability Assessment Schedule (WHODAS 2.0) [15]
- Food frequency questionnaire (Food-Frequency-Questionnaire) [16]
- Physical Activity Questionnaire (BSA-F) [17]

The comprehensibility of the questionnaire will be checked in advance by a cognitive pretest on 8–10 persons and appropriate adjustments will be made if necessary.

#### WP 3: Deliverables

First, the programs identified in WP 1 will be compared with the preferences of the subgroups identified in WP 2 in order to find out whether target group-specific programs already exist. Taking into account all the findings, the next step will be the extraction of components for optimized programs for those for which no suitable programs could be identified in the health-care. Subsequently, lifestyle interventions will be designed that correspond to the specifics of the subgroups. For this purpose, a workshop will be held in which all project partners as well as the Advisory Board (including hepatologists, psychologists, sports scientists, nutritionists, representatives of the health insurance companies) and the Patient Advisory Council will participate. The aim is to derive components that facilitate the target group-specific integration of the patients into the identified and designed lifestyle interventions and to derive components that can guarantee the continuous participation of the subgroups. In the end, an intervention manual is to be developed that will help physicians of specialist practices and outpatient clinics both to steer their patients into a subgroup-specific intervention and to enable their patients to successfully implement the lifestyle intervention.

#### Study population and recruitment

The target population will consist of people with NASH (ICD: K75.8) who have at least one liver fibrosis (severity F2-4), aged 18 years and older, who are being treated in certified specialist practices or hospital outpatient clinics. The recruitment for the online survey will be done by the selected practices and outpatient clinics and. The target region is defined as the locations of those and their surroundings, distributed all over Germany. So far, nine certified specialist practices or hospital outpatient clinics have agreed to participate in the recruitment. This number can be expanded in the course of the project.

Patients either will receive an invitation to the online survey from their physician during a medical appointment, or, if they do not have an appointment during the field phase, be invited directly by mail from the certified specialist practices or hospital outpatient clinics. The invitation will contain information about the study and a link to the online survey. In order to ensure that each patient can only participate once, the invitation will contain a random code, which the patients must enter as a login before the survey begins. This code will be anonymous and can only be assigned to the respective practices or outpatient clinics, not to the individual patients.

#### Sample size calculation

The number of cases in the focus group discussions will be determined by the purposeful sampling method. Initially, 4 focus group discussions with 8 participants each are planned, corresponding to a sample size of n = 32.

For a DCE, optimal sample size planning is guided by the formula according to Johnson [18] & Orme [19], which calculates the number of persons to be interviewed (n) as a function of the number of choice decisions (t), the number of choice alternatives per choice set (a) and the highest number of levels within all attributes (c): n ≥ 1000 * c / t*a. A value of 1,000 is considered desirable, with a value of 500 already considered sufficient. Subject to the attributes and levels selected after systematic review and focus group discussions, a target minimum sample size of n = 250 results from c = 3, t = 6 and a = 2. Since subgroup comparisons between patients with versus without lifestyle intervention experience as well as by gender are to be made, n increases to n = 500. As such, 2,000 patients will be contacted, with an estimated response rate of 25%. A reminder is planned after two and four weeks to increase the response. In addition, advertising materials (flyers) will be displayed in the certified specialist practices and hospital outpatient clinics in order to draw the patients’ attention to the project and increase their motivation to participate.

#### Data analysis

The focus group discussions will be recorded and transcribed by a transcription agency in compliance with data protection guidelines. The qualitative analysis of the transcripts will be carried out using MAXQDA software. The content structuring analysis of the transcribed focus group discussions will be carried out according to Mayring [20].

For the quantitative data, descriptive analyses will be first performed after data clearing. Cluster analyses can provide indications of groups with similar preference patterns. In the final analyses, all respondents who completed the questionnaire in full and answered the dominant choice set correctly will be included. Data will be analyzed using McFadden’s Random Utility Theory [21]. Multivariate statistical methods will be used to analyses the collected data and estimate the choice probabilities or part-worth values. We will start with a classical conditional logit (CL) model to determine the preferences over the whole sample, as well as to check whether the attribute levels have the expected signs. CL models can only identify the best or most preferred alternative in a choice set, while preference heterogeneity within the sample remains undetected. For this reason, a mixed logit (MXL) model will additionally be computed. An MXL model identifies attributes that differ between individuals without being able to explain this preference heterogeneity more precisely. As more precise analysis of preference heterogeneity has recently been made possible with Latent Class (LC) models, an LC model will be computed as well. Here, similar choices of respondents are classified into a latent given number of classes. While preferences are largely homogeneous within a class, preferences differ between classes and thus across the sample. Data analysis will be performed with the statistical program STATA version 18.

#### Ethical considerations

This study was approved by the Ethics Committee of the Hannover Medical School on 20.02.2023 (reference number 10771_BO_K_2023). If necessary, the Ethics Committee will seek approval for changes to the original study protocol. The study will be conducted in accordance with the principles of the Declaration of Helsinki [22]. The principles of "Good Clinical Practice" and all relevant legal, ethical and data protection principles will be observed [23]. All persons actively participating in the study will be comprehensively and understandably informed about the purpose and procedure of the project and the handling of the data collected. Where necessary, the research team obtains informed consent for the qualitative (written consent) and quantitative (online consent) research approaches in compliance with legal, ethical and data protection principles. Participation in the study is voluntary and can be revoked at any time. Non-participation has no consequences. The data will be deleted ten years after the end of the project.

## Discussion

In Germany, a considerable number of people are affected by NASH. The results of the project will provide an overview of the needs and preferences of this patient collective as well as of existing specific care offers. In the clinical context, the research project aims to improve care by further developing sustainable lifestyle intervention services and optimizing them according to the patient’s preferences. Currently, there is no approved medication for the treatment of NASH. It is to be expected that the currently investigated drugs are not effective for all pathogenetically relevant parameters and stages of the disease (metabolism, inflammation, fibrosis) and probably also lead to a considerable economic burden on the health care system due to continuous use [24]. Furthermore, lifestyle interventions will be the most important component of any medical recommendation [2].

The research project will contribute on various levels to the development of knowledge that is not yet available but can form the basis for sustainably optimized care for NASH patients and disease groups to which the knowledge can be transferred (e.g., metabolic syndrome). Based on the project results, a prevention or care offer will be developed that is oriented towards the preferences, needs and abilities of the patients. At the same time, there will be considerable synergy effects in the treatment of common comorbidities such as obesity or DM2. By involving health insurance companies, service providers and health care actors from the various disciplines as well as other stakeholders (e.g., medical and sports associations), the implementation of the newly developed care approach can be planned at an early stage so that a pilot phase to test its effectiveness can follow immediately after the end of this project.

## Supporting information

S1 Checklist STROBE statement—checklist of items that should be included in reports of observational studies. (DOCX)
