# Supplementary material for: Lifestyle interventions for patients with non-alcoholic steato-hepatitis–Design, rationale and protocol of the study “target group-specific optimisation of lifestyle interventions for behavior change in non-alcoholic steato-hepatitis (OPTI-NASH)”
Source: PLoS One. 2023 Jul 27;18(7):e0288905. doi: 10.1371/journal.pone.0288905 (PMC10374068; doi:10.1371/journal.pone.0288905)
Supplement: S1 Checklist — (DOCX) [file pone.0288905.s001.docx]

STROBE Statement—checklist of items that should be included in reports of observational studies

|  | Item No. | Recommendation | Page  No. | Relevant text from manuscript |
| --- | --- | --- | --- | --- |
| **Title and abstract** | 1 | (*a*) Indicate the study’s design with a commonly used term in the title or the abstract | p. 2 | “An online survey will be used to elicit patient’s preferences on program design and on motivational aspects in a cross-sectional design.” |
|  |  | (*b*) Provide in the abstract an informative and balanced summary of what was done and what was found | p. 2 | “Based on a systematic review and focus group discussions, two discrete choice experiments (DCE) will be designed, one on aspects influencing successful uptake of lifestyle interventions and one to analyses parameters contributing to long-term participation. An online survey will be used to elicit patient’s preferences on program design and on motivational aspects in a cross-sectional design. The recruitment will take place in nine certified specialist practices and hospital outpatient clinics aiming to reach a sample size of n = 500 which is also required for the DCE design.” |
| Introduction | | | |  |
| Background/rationale | 2 | Explain the scientific background and rationale for the investigation being reported | p. 3-4 | *See introduction.* |
| Objectives | 3 | State specific objectives, including any prespecified hypotheses | p. 4-5 | *See study aim and setting.* |
| Methods | | | |  |
| Study design | 4 | Present key elements of study design early in the paper | p. 5-6;  Figure 1 | “The project is divided into three WP according to the research questions: (1) First, lifestyle interventions are researched based on a scoping review and a screening of current available intervention programs. Characteristics of effective programs that have high participation rates and low dropout rates will be extracted. (2) Based on this, two Discrete Choice Experiments (DCE) will be developed, one on program design and one on motivation. In an online survey, knowledge, motivation, experiences, and preferences of a representative patient sample will be quantitatively assessed. The DCEs serve to examine which lifestyle interventions are preferred by the patients and which aspects are relevant to decision-making. (3) In the last step, recommendations for components of an optimized lifestyle change program, which takes into account the preferences of the subgroups and the clinical context from a hepatological perspective, will be derived from the generated data. Components will be defined that facilitate the target group-specific integration of patients into programs as well as that ensure the continuous participation of the subgroups.” |
| Setting | 5 | Describe the setting, locations, and relevant dates, including periods of recruitment, exposure, follow-up, and data collection | p. 11-12 | *See study population and recruitment.* |
| Participants | 6 | (*a*) *Cohort study*—Give the eligibility criteria, and the sources and methods of selection of participants. Describe methods of follow-up  *Case-control study*—Give the eligibility criteria, and the sources and methods of case ascertainment and control selection. Give the rationale for the choice of cases and controls  *Cross-sectional study*—Give the eligibility criteria, and the sources and methods of selection of participants | p.11-12 | “The target population will consist of people with NASH (ICD: K75.8) who have at least one liver fibrosis (severity F2-4), aged 18 years and older, who are being treated in certified specialist practices or hospital outpatient clinics.” |
|  |  | (*b*) *Cohort study*—For matched studies, give matching criteria and number of exposed and unexposed  *Case-control study*—For matched studies, give matching criteria and the number of controls per case | n/a |  |
| Variables | 7 | Clearly define all outcomes, exposures, predictors, potential confounders, and effect modifiers. Give diagnostic criteria, if applicable | n/a |  |
| Data sources/ measurement | 8* | For each variable of interest, give sources of data and details of methods of assessment (measurement). Describe comparability of assessment methods if there is more than one group | p. 7-11 | *See WP 2: Online survey including DCE.* |
| Bias | 9 | Describe any efforts to address potential sources of bias | n/a |  |
| Study size | 10 | Explain how the study size was arrived at | p. 12-13 | *See sample size calculation.* |

Continued on next page

| Quantitative variables | 11 | Explain how quantitative variables were handled in the analyses. If applicable, describe which groupings were chosen and why | p. 13 | *See Data analysis.* |
| --- | --- | --- | --- | --- |
| Statistical methods | 12 | (*a*) Describe all statistical methods, including those used to control for confounding | p. 13 | *See Data analysis.* |
|  |  | (*b*) Describe any methods used to examine subgroups and interactions | p. 13 | “Multivariate statistical methods will be used to analyses the collected data and estimate the choice probabilities or part-worth values. We will start with a classical conditional logit (CL) model to determine the preferences over the whole sample, as well as to check whether the attribute levels have the expected signs. CL models can only identify the best or most preferred alternative in a choice set, while preference heterogeneity within the sample remains undetected. For this reason, a mixed logit (MXL) model will additionally be computed. An MXL model identifies attributes that differ between individuals without being able to explain this preference heterogeneity more precisely. As more precise analysis of preference heterogeneity has recently been made possible with Latent Class (LC) models, an LC model will be computed as well. Here, similar choices of respondents are classified into a latent given number of classes. While preferences are largely homogeneous within a class, preferences differ between classes and thus across the sample.” |
|  |  | (*c*) Explain how missing data were addressed | p. 13 | “In the final analyses, all respondents who completed the questionnaire in full and answered the dominant choice set correctly will be included.” |
|  |  | (*d*) *Cohort study*—If applicable, explain how loss to follow-up was addressed  *Case-control study*—If applicable, explain how matching of cases and controls was addressed  *Cross-sectional study*—If applicable, describe analytical methods taking account of sampling strategy | n/a |  |
|  |  | (*e*) Describe any sensitivity analyses | n/a |  |
| Results | | | | |
| Participants | 13* | (a) Report numbers of individuals at each stage of study—eg numbers potentially eligible, examined for eligibility, confirmed eligible, included in the study, completing follow-up, and analysed | n/a |  |
|  |  | (b) Give reasons for non-participation at each stage | n/a |  |
|  |  | (c) Consider use of a flow diagram | n/a |  |
| Descriptive data | 14* | (a) Give characteristics of study participants (eg demographic, clinical, social) and information on exposures and potential confounders | n/a |  |
|  |  | (b) Indicate number of participants with missing data for each variable of interest | n/a |  |
|  |  | (c) *Cohort study*—Summarise follow-up time (eg, average and total amount) | n/a |  |
| Outcome data | 15* | *Cohort study*—Report numbers of outcome events or summary measures over time | n/a |  |
|  |  | *Case-control study—*Report numbers in each exposure category, or summary measures of exposure | n/a |  |
|  |  | *Cross-sectional study—*Report numbers of outcome events or summary measures | n/a |  |
| Main results | 16 | (*a*) Give unadjusted estimates and, if applicable, confounder-adjusted estimates and their precision (eg, 95% confidence interval). Make clear which confounders were adjusted for and why they were included | n/a |  |
|  |  | (*b*) Report category boundaries when continuous variables were categorized | n/a |  |
|  |  | (*c*) If relevant, consider translating estimates of relative risk into absolute risk for a meaningful time period | n/a |  |

Continued on next page

| Other analyses | 17 | Report other analyses done—eg analyses of subgroups and interactions, and sensitivity analyses | n/a |  |
| --- | --- | --- | --- | --- |
| Discussion | | | | |
| Key results | 18 | Summarise key results with reference to study objectives | n/a |  |
| Limitations | 19 | Discuss limitations of the study, taking into account sources of potential bias or imprecision. Discuss both direction and magnitude of any potential bias | n/a |  |
| Interpretation | 20 | Give a cautious overall interpretation of results considering objectives, limitations, multiplicity of analyses, results from similar studies, and other relevant evidence | n/a |  |
| Generalisability | 21 | Discuss the generalisability (external validity) of the study results | n/a |  |
| Other information | |  | | |
| Funding | 22 | Give the source of funding and the role of the funders for the present study and, if applicable, for the original study on which the present article is based | p. 16 | “This study is funded (on the basis of a peer review) by the German Federal Joint Committee’s Innovation Fund (grant number 01VSF22032) from 1 January 2023 to 31 December 2025. The funders did not and will not have a role in study design, data collection and analysis, decision to publish, or preparation of the manuscript.” |
|  |  |  |  |  |

*Give information separately for cases and controls in case-control studies and, if applicable, for exposed and unexposed groups in cohort and cross-sectional studies.

**Note:** An Explanation and Elaboration article discusses each checklist item and gives methodological background and published examples of transparent reporting. The STROBE checklist is best used in conjunction with this article (freely available on the Web sites of PLoS Medicine at http://www.plosmedicine.org/, Annals of Internal Medicine at http://www.annals.org/, and Epidemiology at http://www.epidem.com/). Information on the STROBE Initiative is available at www.strobe-statement.org.
