# Supplementary material for: Lifestyle interventions for patients with non-alcoholic steato-hepatitis–Design, rationale and protocol of the study “target group-specific optimisation of lifestyle interventions for behavior change in non-alcoholic steato-hepatitis (OPTI-NASH)”
Source: PLoS One. 2023 Jul 27;18(7):e0288905. doi: 10.1371/journal.pone.0288905 (PMC10374068; doi:10.1371/journal.pone.0288905)
Supplement: Peer review history

# Editor report: Decision Letter 0

**DOI:** 10.1371/journal.pone.0288905.r001

**Authors:** Richard Ali

6 Jul 2023

Lifestyle interventions for patients with non-alcoholic steato-hepatitis – Design, rationale and protocol of the study “Target group-specific optimisation of lifestyle interventions for behavior change in non-alcoholic steato-hepatitis (OPTI-NASH)”

PONE-D-23-19463

Dear Dr. Krüger,

We’re pleased to inform you that your manuscript has been judged scientifically suitable for publication and will be formally accepted for publication once it meets all outstanding technical requirements.

Within one week, you’ll receive an e-mail detailing the required amendments. When these have been addressed, you’ll receive a formal acceptance letter and your manuscript will be scheduled for publication.

An invoice for payment will follow shortly after the formal acceptance. To ensure an efficient process, please log into Editorial Manager at http://www.editorialmanager.com/pone/, click the 'Update My Information' link at the top of the page, and double check that your user information is up-to-date. If you have any billing related questions, please contact our Author Billing department directly at authorbilling@plos.org.

If your institution or institutions have a press office, please notify them about your upcoming paper to help maximize its impact. If they’ll be preparing press materials, please inform our press team as soon as possible -- no later than 48 hours after receiving the formal acceptance. Your manuscript will remain under strict press embargo until 2 pm Eastern Time on the date of publication. For more information, please contact onepress@plos.org.

Kind regards,

Richard Ali, PhD

Staff Editor

PLOS ONE

Journal requirements:

When submitting your revision, we need you to address these additional requirements.

1. Please ensure that your manuscript meets PLOS ONE's style requirements, including those for file naming. The PLOS ONE style templates can be found at

https://journals.plos.org/plosone/s/file?id=wjVg/PLOSOne_formatting_sample_main_body.pdf and

https://journals.plos.org/plosone/s/file?id=ba62/PLOSOne_formatting_sample_title_authors_affiliations.pdf

2. PLOS requires an ORCID iD for the corresponding author in Editorial Manager on papers submitted after December 6th, 2016. Please ensure that you have an ORCID iD and that it is validated in Editorial Manager. To do this, go to ‘Update my Information’ (in the upper left-hand corner of the main menu), and click on the Fetch/Validate link next to the ORCID field. This will take you to the ORCID site and allow you to create a new iD or authenticate a pre-existing iD in Editorial Manager. Please see the following video for instructions on linking an ORCID iD to your Editorial Manager account: https://www.youtube.com/watch?v=_xcclfuvtxQ

3. Please amend either the title on the online submission form (via Edit Submission) or the title in the manuscript so that they are identical.

# Editor report: Acceptance letter

**DOI:** 10.1371/journal.pone.0288905.r002

**Authors:** Richard Ali

19 Jul 2023

PONE-D-23-19463 

Lifestyle interventions for patients with non-alcoholic steato-hepatitis – Design, rationale and protocol of the study “Target group-specific optimisation of lifestyle interventions for behavior change in non-alcoholic steato-hepatitis (OPTI-NASH)” 

Dear Dr. Krüger:

I'm pleased to inform you that your manuscript has been deemed suitable for publication in PLOS ONE. Congratulations! Your manuscript is now with our production department. 

If your institution or institutions have a press office, please let them know about your upcoming paper now to help maximize its impact. If they'll be preparing press materials, please inform our press team within the next 48 hours. Your manuscript will remain under strict press embargo until 2 pm Eastern Time on the date of publication. For more information please contact onepress@plos.org.

If we can help with anything else, please email us at plosone@plos.org. 

Thank you for submitting your work to PLOS ONE and supporting open access. 

Kind regards, 

PLOS ONE Editorial Office Staff

on behalf of

Richard Ali 

Staff Editor

PLOS ONE
